# Associations between problematic smartphone use and behavioural difficulties, quality of life, and school performance among children and adolescents

**DOI:** 10.1186/s12888-022-03815-4

**Published:** 2022-03-18

**Authors:** Tobias Kliesener, Christof Meigen, Wieland Kiess, Tanja Poulain

**Affiliations:** 1 LIFE Leipzig Research Center for Civilization Diseases, Leipzig University, Philipp-Rosenthal-Strasse 27, 04103 Leipzig, Germany; 2 Department of Women and Child Health, University Hospital for Children and Adolescents and Center for Pediatric Research, Leipzig University, Liebigstrasse 20a, 04103 Leipzig, Germany

**Keywords:** Problematic smartphone use, PSU, Children, Behavioural addiction, Quality of life, Behavioural difficulties, School performance

## Abstract

**Background:**

European studies on determinants and factors associated with problematic smartphone use (PSU) in children and adolescents are still sparse. This study reports the current amount of PSU symptoms and the presence of (clinically relevant) PSU in German children and adolescents. We also investigated associations between socio-demographic factors, different smartphone usage patterns, and daily smartphone usage time and the amount of PSU symptoms in this group. In addition, associations of PSU symptoms and high smartphone usage times (> 2 h/day) with behavioural problems, quality of life (QoL), and school performance were investigated.

**Methods:**

Within the framework of the LIFE Child study, 564 children and adolescents aged 10–18 years provided information on PSU symptoms (using the Smartphone Addiction Proneness Scale), daily smartphone usage time, smartphone activities, behavioural strengths and difficulties (using the Strengths and Difficulties Questionnaire), QoL (using the KIDSCREEN-27), and school performance. Multiple regression analyses were applied to assess associations.

**Results:**

In the present sample, PSU was present in 13 children (2.3%). Older age, female gender, high daily smartphone usage time of > 2 h, and intensive smartphone use for social networking, gaming, or watching video clips were significantly associated with more PSU symptoms. Children and adolescents reporting more PSU symptoms also showed lower QoL, more behavioural difficulties, and poorer school performance, independently of age, gender, socio-economic status, and daily smartphone usage time. In contrast, daily smartphone usage time per se showed only weak or non-significant associations with these aspects of health and behaviour.

**Conclusion:**

Intensive smartphone use for entertainment may increase the risk of developing PSU symptoms. Furthermore, the results indicate that PSU symptoms (more than long smartphone usage times per se) are associated with more behavioural difficulties and poorer QoL.

## Background

Mobile media, especially smartphones, have revolutionized media behaviour. The smartphone is a core possession for most people, [1] and its use among children and adolescents has increased dramatically in the last 10 years [2–4]. In Germany, 93% of 12- to 19-year-olds own a smartphone, and 92% state that they use their smartphone on a daily basis. On average, 12- to 19-year-old children and adolescents in Germany spent around 205 min online per day, i.e., using the internet on different media devices [5]. In recent years, it has become clear that heavy Internet use, such as playing computer games online or using social networks, can be associated with symptoms similar to those of behavioural addictions such as gambling [6–8]. Gaming Disorder has been included as a diagnosis in the International Classifications of Diseases and Related Health Problems (ICD-11) [9]. In the Diagnostic and Statistical Manual of Mental Disorders (DSM-5), Internet Gaming Disorder has been included as a condition warranting further study [10]. As smartphones provide (unlimited) access to the Internet, concerns about “smartphone addiction” have also arisen [6, 11]. However, the term “smartphone addiction” is controversial and difficult to differentiate from other technology-related addictions (e.g., gaming disorder, internet addiction). The smartphone is a physical object whose features – portability, speed and privacy – might contribute to excessive use, loss of control, and other symptoms typical of behavioural addiction. However, problems that arise from smartphone use are likely to be related to the nature of the activity, motivation, or gratification with which it is used, not the smartphone per se [8]. Moreover, the characteristics of excessive smartphone use might not sufficiently fulfill the criteria of an addiction. Finally, “smartphone addiction” is not listed as a diagnosis in DSM or ICD. As recommended in a previous review [8], we will therefore use the term “problematic smartphone use” (PSU) instead of “smartphone addiction” to refer to smartphone use associated with symptoms of behavioural addiction. By taking into account symptoms of behavioural addiction, PSU can be distinguished from pure smartphone usage time, which disregards these aspects. At the same time, previous studies show strong associations between longer smartphone usage times and PSU symptoms [12, 13], indicating that excessive use may be at least a part of PSU. Several studies [14–16] have investigated associations between PSU and socio-demographic parameters such as gender, age, or socio-economic status (SES). Until now, there is disagreement about whether girls or boys are more likely to exhibit PSU. Studies that specifically examined the influence of gender were unable to detect a difference between sexes [14, 17]. However, Lee et. al and Park et. al showed more problematic smartphone use in girls than in boys [18, 19]. With older children having more frequent access to smartphones [5], studies have shown that among children and adolescents, an older age is associated with more PSU symptoms [14, 20]. A family’s SES, usually captured by parents’ education, income and occupation, has been shown to be associated with child health and behaviour [21]. Regarding the use of electronic media, previous studies have shown generally higher media use in children and adolescents from lower compared to higher social strata [16, 22, 23]. However, it is still unclear whether the same trend can be observed regarding PSU, i.e., a phenomenon characterized by more than just long usage times. In a previous study, SES was not associated with PSU, suggesting that PSU might occur in all social strata [24]. Regarding possible associations between PSU and children and adolescents’ health, previous studies reported negative associations with psychological well-being [25–27], mood [28], prosocial behaviour [29], peer relations [29], and academic performance [30] as well as positive associations with behavioural and emotional problems [31]. For excessive smartphone use, i.e., long usage times without considering symptoms of behavioural addiction, associations with poorer sleep quality [32], bad mood [33], low psychological well-being [34] and behavioural and emotional problems [35] were observed. It is important to mention that most previous studies on PSU were performed in Asian countries (especially South Korea [36]) and in older adolescents or young adults [15, 17, 28]. They reported that a high number of adolescents (mean age 15.6 years) in South Korea showed PSU (30.9%) [13]. However, the presence of PSU or amount of PSU symptoms might differ between European and Asian children and adolescents and within European countries as well, e.g., due to differences in cultural norms or digitalisation. In Asian countries, it is more difficult to freely socialize, which may contribute to a higher smartphone usage [8]. Also, technology absorption is much more advanced in Asia compared to Europe. For example, in 2015, South Korea had the highest smartphone ownership rate (88%) worldwide [13]. European studies indicate a lower rate of PSU than in South Korea, e.g., 9% in Spain [37] and 10% in the UK [38]. However, a direct comparison of prevalence rates obtained in different studies is difficult because of differences in methods and instruments used. As exposure to and ownership of smartphones among children and adolescents have increased in Germany over the last few years [3, 5], it is important to investigate PSU not only in older adolescents, but also in children, as both groups experience fundamental development challenges that affect them in many ways (e.g., development of a self-concept and self-awareness, recognition by peers and family members, control of emotions, development of sexuality, and desire for independence [39]). The first aim of the present study was to determine the current presence of PSU in German children and adolescents. We expected a number similar to other European studies but lower than previously reported in Asian youth. Furthermore, we explored whether long smartphone usage times and specific smartphone usage patterns (e.g., intensive social networking or intensive gaming) are associated with PSU symptoms. Based on previous studies [12, 13], we expected significant associations with long usage times and, in particular, with intensive social networking [40, 41]. Another aim was to investigate associations between PSU symptoms and long smartphone usage times (on the one hand) and socio-demographic factors such as age, sex, and SES (on the other hand). In line with previous studies [18, 20], we expected more PSU symptoms and longer smartphone usage times in older versus younger children and in girls versus boys. With regard to SES, we assumed that children and adolescents growing up in families with a lower SES would report longer smartphone usage times. Regarding PSU symptoms, we did not expect a difference between social classes. Finally, we aimed to assess associations between PSU symptoms and smartphone usage times and QoL, behavioural difficulties and school performance. We hypothesized positive associations with behavioural difficulties and negative associations with QoL and school performance. Given that PSU symptoms are defined to consider symptoms of behavioural addiction, we expected to observe stronger associations with PSU than with mere smartphone usage time.

## Methods

### Participants

The analysed data were collected between 2018 and 2020 in the LIFE Child Study (LIFE – Leipzig Research Center for Civilization Diseases, University of Leipzig) [42, 43]. The LIFE Child study is a cohort study investigating healthy child development and the development of civilization diseases. Participants are mainly recruited via advertisement at different institutions, such as hospitals and public health centers. The first study visit takes place when the child is between 0 and 16 years old, and subsequent visits are scheduled every year. The study program consists of multiple examinations, tests and biological samples conducted by trained study assistants, as well as questionnaires that are completed by parents or the children and adolescents themselves. Information relevant to the present study was provided by 864 children and adolescents at different study visits, resulting in a total of 1537 completed questionnaires. If a child participated in the study at more than one time point (*n* = 673), only the last visit was considered. Furthermore, all children and adolescents not owning a smartphone were excluded (*n* = 65). Information on smartphone use, QoL, school performance, behavioural difficulties, and SES was available for 564 of these children and adolescents, leading to the final sample (287 male, 277 female, mean age = 13.76, age range = 10–18). Informed written consent was provided by all parents and 18-year-old children before inclusion in the study. The study was conducted in accordance with the Declaration of Helsinki, and the study protocol was approved by the Ethics Committee of the University of Leipzig (Reg. No. 264–10-19,042,010).

## Measures

### Problematic smartphone use (PSU)

PSU symptoms were assessed using the German version of the Smartphone Addiction Proneness Scale (SAPS) [44]. This questionnaire was completed by the children and adolescents themselves and consists of 15 items capturing four subdomains (adaptation, virtual existence, withdrawal, and control). Responses are given on a four-point Likert scale ranging from 1 (no agreement) to 4 (full agreement). Scores on single items are summed up to a total score ranging from 15 to 54, with higher scores indicating more PSU symptoms. This score was used for further analysis. The previously proposed cut-off of 42 was used to identify children showing (clinically) relevant PSU [13]. In the present sample, Cronbach's alpha for the total score was 0.84. The validity of the SAPS has been tested in other studies [44, 45].

### Smartphone usage duration and intensive use of various smartphone activities

Smartphone usage time was assessed in a self-report questionnaire created by the authors that had already been applied in other research projects [46, 47]. In this questionnaire, children and adolescents are asked to estimate the amount of time they use their smartphone (online and offline) on a typical weekday and a typical weekend day. For each question, participants have to choose the most appropriate of five response options (never, approximately 30 min, 1–2 h, 3–4 h, > 4 h). For further analysis, the responses were transformed into hours of daily smartphone use (0, 0.5, 1.5, 3.5, 5*).* Responses to the four separate questions were combined to create a new variable capturing daily smartphone usage time (((online-weekday + offline-weekday) *5) + (online-weekend + offline-weekend) *2)/7). Finally, all daily smartphone usage times longer than 2 h (> 2 h/d) were classified as high smartphone use. This cut-off was chosen based on current recommendations to limit daily screen time to a maximum of 2 h [4, 48, 49]. Shorter usage times were classified as low/normal. The questionnaire on smartphone usage time also contains questions on the frequency (never, sometimes, or often) of different smartphone activities (watching video clips, playing games, writing text messages, social networking, searching for information). Engagement in an activity was categorized as intensive if the participant reported performing the activity “often” and additionally reported a “high” daily smartphone usage time.

### Behavioural strengths and difficulties

The self-report version of the Strengths and Difficulties Questionnaire (SDQ) was used to assess behavioural strengths and difficulties [50, 51]. The five scales cover the fields of prosocial behaviour, hyperactivity/inattention, emotional problems, conduct problems, and peer relationship problems. Each scale consists of five questions. Answers are given on a three-point scale (0 = not true, 1 = somewhat true, 2 = certainly true). For each scale, scores on the individual questions are summarized into a sum score, with higher scores indicating more difficulties or – in the case of prosocial behaviour – more strengths. These sum scores were used for further analysis. In the present sample, Cronbach`s alpha was 0.66, 0.70, 0.73, 0.66 and 0.71 for the prosocial behaviour, hyperactivity, emotional problems, conduct problems, and peer relationship problems scales, respectively.

### Quality of life (QoL)

QoL was assessed using the self-report version of the KIDSCREEN-27 [52]. This questionnaire consists of 27 health-related questions in the areas of physical well-being, psychological well-being, parent relationship/home life, social support/peer relationships and the school environment. Responses are given on a five-point Likert scale. Scores on single items are summed up into scale-specific sum scores. These sum scores are then transformed into gender- and age-specific t-values (mean = 50, sd = 20). These values were used for further analysis. In the present sample, Cronbach`s alpha was 0.79, 0.87, 0.80, 0.88, 0.82 for the physical well-being, psychological well-being, parent relationship/home life, social support/peers, and school environment scales, respectively.

### School performance

Information on school grades in the subjects German (= language of instruction), mathematics, physical education, and first foreign language were provided by children and adolescents themselves. According to the German grading system, a grade of 1 reflects top performance, while 6 reflects the worst performance. For further analysis, grades of 1–2 were categorized as “good” and grades of 3–6 (of which 5 and 6 were rarely reported) were categorized as “poor”.

### Socio-economic status (SES)

SES was captured by a composite score (adapted from [53, 54]) considering the education (academic and professional education) and occupational status of the participants’ mother and father as well as the monthly household disposable income. Information was provided by the participants’ parents. Scores range from 3 to 21, with higher values reflecting a higher SES. Based on cut-offs created in a representative sample for the German population [55], these scores can be used to categorize SES as either low (3–8.4 points), middle (8.5–15.4 points), or high (15.5–21 points). In a representative sample, the distribution of low, middle, and high SES should be approximately 20%, 60%, and 20% [55].

### Statistical analysis

The analyses were conducted using R 4.0.3. Associations between PSU symptoms (indicated by higher SAPS scores) and daily smartphone usage times (high versus low/normal) or intensive use of different smartphone activities were assessed using linear regression analyses, with PSU symptoms included as dependent variable. Associations of PSU symptoms and long smartphone usage times with socio-demographic parameters were assessed using multiple linear or logistic regression analyses. Age, gender and SES were included (simultaneously) as independent variables, and either PSU symptoms or daily smartphone usage time (high versus low/normal) was included as dependent variable.

Associations of PSU and long smartphone usage times with behavioural difficulties, QoL, and school performance were assessed using linear or logistic regression analyses. Behavioural difficulties (indicated by scores on the individual SDQ scales), QoL (indicated by scores on the individual KIDSCREEN-17 scales), or school performance were included as dependent variables. In a first step (simple regression analyses), we applied two separate models, with either PSU symptoms or daily smartphone use time as independent variable. In a next step (multiple regression), both were included simultaneously in the same model. All associations were adjusted for age, gender and SES (as a continuous measure). A *p*-value < 0.05 was considered to indicate a significant association. For each regression model, we checked the model quality by visual inspection using different diagnostic plots (residual plot vs. fitted and Q-Q-plot). The residuals were independent of the fitted values, sufficiently normally distributed, and revealed sufficiently stable variance across the full range of the dependent variable.

## Results

### Descriptive analysis

The majority of participants (*n* = 340 (60%)) were categorized to the middle SES group, 205 (36%) to the higher SES group, and 19 (4%) to the lower SES group. Of the 564 children and adolescents analysed, 374 (66,3%) reported a high daily smartphone usage time > 2 h/d, while *n* = 190 (33,7%) remained below this mark. The frequencies of different smartphone activities are shown in Table 1. Participants most frequently used their smartphones for social networking (*n* = 277 (49%) intensive usage). The mean total SAPS score was 27.8 (sd = 6.23, min. = 15.0, max. = 54.0), and 13 (2.3%) participants scored higher than 42, indicating the presence of (clinically relevant) PSU. In male participants, the mean SAPS score was 26.9, and 1.74% showed (clinically relevant) PSU. In female participants, the mean total SAPS score was 28.7, and 2.88% reported (clinically relevant) PSU.

**Table 1:** Associations of high daily smartphone usage time (> 2 h/d) and intensive use of different smartphone activities with SAPS scores (PSU symptoms) (*N* = 564)

	**dependent variable**							
**Independent variables**	**SAPS score (PSU symptoms)**							
**high daily smartphone usage time (> 2 h/d)**	**n**	**b**	**CI 95%**	**ß**	**t**	**p**	R^2^	F
	374	**2.81**	1.61 – 4.00	0.20	4.61	< .001	0.08	< .001
**intensive use of smartphone activities (frequent usage + high daily smartphone usage time)**								
social networking	277	**3.65**	2.50 – 4.80	0.27	6.24	< .001	0.11	< .001
writing text messages	140	1.24	-0.03 – 2.50	0.08	1.91	0.05	0.05	< .001
watching video clips	190	**2.06**	0.92 – 3.20	0.15	3.56	< .001	0.07	< .001
gaming	144	**2.39**	1.15 – 3.63	0.16	3.79	< .001	0.07	< .001
searching for information	94	1.25	-0.22 – 2.72	0.07	1.67	0.10	0.05	< .001

All associations are adjusted for gender, age, SES, *b* non-standardized regression coefficient; *ß* standardized regression coefficient. Significant associations are highlighted in bold

### Associations of SAPS scores (PSU symptoms), daily smartphone usage time and smartphone activities

**Fig. 1:**
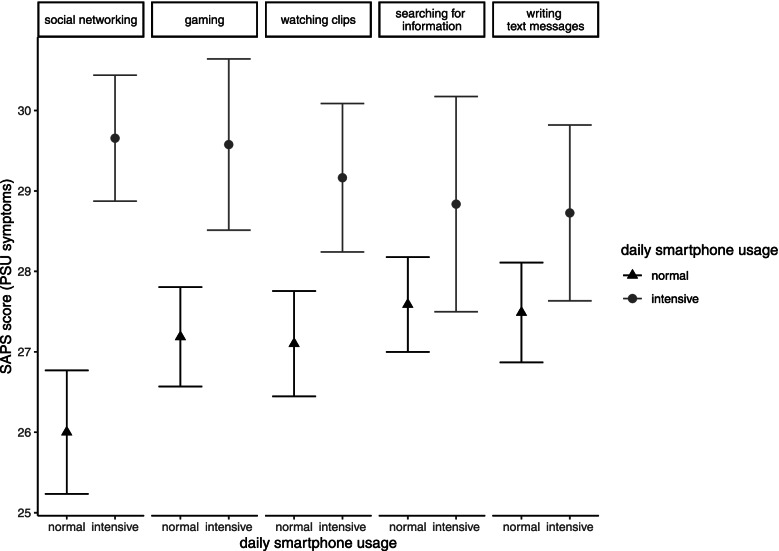
Effect plots illustrating the associations between SAPS scores (PSU symptoms) and daily engagement in different smartphone activities, divided into normal and intensive usage. Intensive usage is indicated by total smartphone usage time > 2 h/d and high frequency of the specific activity

Children and adolescents reporting that they used their smartphone > 2 h/d exhibited significantly higher SAPS scores than children and adolescents reporting shorter daily smartphone usage durations. (b = 2.81, *p* < 0.001, see Table 1 and Fig. 1). Regarding different smartphone activities, intensive gaming, intensive video watching, and intensive social networking were significantly associated with higher SAPS scores (b = 2.39, 2.06, and 3.65, respectively, all *p* < 0.001). In contrast, intensive searching for information or sending text messages (SMS) were not significantly associated with higher SAPS scores (see Table 1).

### Associations of SAPS scores (PSU symptoms) and daily smartphone usage time with age, gender and SES

Higher age was significantly associated with higher SAPS scores (indicating more PSU symptoms; (b = 0.56, *p* < 0.001)) and with smartphone usage times > 2 h/d (OR = 1.35, *p* < 0.001) (see Table 2). For 10-year-old children, the likelihood of usage times > 2 h/d was estimated at 42%, compared to 89% in 18-year-olds. Similarly to an older age, female gender was significantly associated with higher SAPS scores (b = 1.89, *p* < 0.001) and smartphone usage times > 2 h/d (OR = 1.75, *p* < 0.01). 75% of girls were estimated to have a smartphone usage time > 2 h/d, compared to 63% of boys. Regarding SES, smartphone usage times > 2 h/d were significantly more frequent in children and adolescents with a lower familial SES (OR = 0.83, p < 0.001). However, we did not find a significant association between SES and PSU symptoms.

**Table 2:** Associations of age, gender, and SES with SAPS scores (PSU symptoms) and high smartphone usage time (> 2 h/d) (*N* = 564)

dependent variables				
		**SAPS score (PSU symptoms)**		**high daily smartphone usage time (> 2 h/d)**
**independent variables**	**n**	564	**n**	564
**age**	**b**	**0.56**	**OR**	**1.35**
	**CI 95%**	0.28 – 0.84	**CI 95%**	1.22 – 1.50
	**ß**	0.16	**p**	< .001
	**t**	3.92		
	**p**	< .001		
**gender(female)**	**b**	**1.89**	**OR**	**1.75**
	**CI 95%**	0.81 – 2.96	**CI 95%**	1.19 – 2.55
	**ß**	0.14	**p**	0.004
	**t**	3.45		
	**p**	< .001		
**SES**	**b**	-0.08	**OR**	**0.83**
	**CI 95%**	-0.25 – 0.09	**CI 95%**	0.78 – 0.89
	**ß**	-0.04	**p**	< .001
	**t**	-0.93		
	**p**	0.35		
	**R**^**2**^	0.05		
	**F**	< .001		

*b* non-standardized regression coefficient, *ß* standardized regression coefficient, *OR* odds ratio. Significant associations are highlighted in bold

### Associations of SAPS scores (PSU symptoms) and daily smartphone usage time with SDQ scores (behavioural difficulties)

The mean scores (+ sd) on the different SDQ scales are displayed in Table 3. In the simple regression analyses, children and adolescents reporting higher SAPS scores also reported higher SDQ scores on the hyperactivity, emotional problems, conduct problems, and peer relationship problems scales, and lower scores on the prosocial behaviour scale (see Table 3). Smartphone usage times > 2 h/d were significantly associated with higher scores on the emotional problems and conduct problems scales (both b = 0.41, *p* < 0.05 and *p* < 0.01, respectively), but not with scores on the other scales. In the multiple regression analysis, all associations between SAPS scores and scores on the SDQ scales remained significant, whereas the associations with smartphone usage times > 2 h/d did not (see Table 3).

**Table 3:** Associations of SAPS scores (PSU symptoms) and high smartphone usage time (> 2 h/d) with SDQ scores (behavioural difficulties) (*N* = 564)

**behavioural difficulties represented by SDQ Scores**						
		**prosocial behaviour**	**hyperactivity**	**emotional problems**	**conduct problems**	**peer relationship problems**
Mean (sd)		7.83 (1.74)	3.50 (2.12)	2.30 (2.21)	1.71 (1.38)	2.14 (1.68)
**Simple Regression**						
SAPS score (PSU symptoms)	**b**	**-0.05**	**0.11**	**0.11**	**0.06**	**0.03**
	**CI 95%**	-0.07—-0.03	0.09 – 0.14	0.08 – 0.13	0.05 – 0.08	0.01 – 0.05
	**ß**	-0.19	0.36	0.33	0.31	0.11
	**t**	-4.59	8.99	8.67	7.68	2.71
	**p**	< .001	< .001	< .001	< .001	0.007
	**R**^**2**^	0.10	0.15	0.22	0.14	0.04
	**F**	< .001	< .001	< .001	< .001	< .001
high smartphone usage time (> 2 h/d)	**b**	-0.19	0.39	**0.41**	**0.41**	0.19
	**CI 95%**	-0.51 – 0.12	-0.001 – 0.78	0.02 – 0.80	0.16 – 0.66	-0.12 – 0.50
	**ß**	-0.05	0.09	0.09	0.14	0.05
	**t**	-1.21	1.96	2.08	3.19	1.19
	**p**	0.22	0.05	0.04	0.001	0.23
	**R**^**2**^	0.07	0.04	0.12	0.06	0.03
	**F**	< .001	< .001	< .001	< .001	0.004
**Multiple Regression**						
SAPS score (PSU symptoms)	**b**	**-0.05**	**0.11**	**0.11**	**0.06**	**0.03**
	**CI 95%**	-0.07—-0.03	0.09 – 0.14	0.08 – 0.13	0.04 – 0.08	0.01 – 0.05
	**ß**	-0.18	0.35	0.33	0.29	0.11
	**t**	-4.43	8.75	8.40	7.20	2.53
	**p**	< .001	< .001	< .001	< .001	0.01
high smartphone usage time (> 2 h/d)	**b**	-0.06	0.07	0.11	0.23	0.11
	**CI 95%**	-0.38 – 0.26	-0.30 – 0.44	-0.27 – 0.48	-0.01 – 0.48	-0.20 – 0.43
	**ß**	-0.02	0.01	0.02	0.08	0.03
	**t**	-0.36	0.37	0.56	1.89	0.69
	**p**	0.71	0.71	0.57	0.06	0.49
	**R**^**2**^	0.10	0.16	0.22	0.14	0.04
	**F**	< .001	< .001	< .001	< .001	< .001

All associations are adjusted for gender, age, SES, *b* non-standardized regression coefficient, *ß* standardized regression coefficient. Significant associations are highlighted in bold

### Associations of SAPS scores (PSU symptoms) and daily smartphone usage time with KIDSCREEN-27 (QoL)

Mean scores (+ sd) on the different scales of the KIDSCREEN-27 are displayed in Table 4. The analyses revealed significant associations between higher SAPS scores and lower scores on all KIDSCREEN-27 scales (see Table 4). These associations remained significant in the multiple regression analyses. In contrast, we observed no significant associations between smartphone usage times > 2 h/d and the KIDSCREEN-27 scales in either the simple or multiple regression analyses.

**Table 4:** Associations of SAPS scores (PSU symptoms) and high smartphone usage time (> 2 h/d) with KIDSCREEN-27 scores (QoL) (*N* = 564)

**QoL represented by KIDSCREEN-27**						
		**physical well-being**	**psychological well-being**	**parent relation/ home life**	**social support/ peers**	**school environment**
Mean (sd)		52.39 (9.66)	51.10 (10.10)	55.98 (10.02)	53.17 (11.16)	54.02 (9.63)
**Simple Regression**						
SAPS score (PSU symptoms)	**b**	**-0.39**	**-0.44**	**-0.40**	**-0.25**	**-0.5**
	**CI**	-0.51—-0.28	-0.56—-0.33	-0.52—-0.28	-0.39—-0.11	-0.61—-0.38
	**95%**	-0.27	-0.29	-0.27	-0.15	-0.34
	**ß**	-6.77	-7.45	-6.54	-3.45	-8.49
	**t**	< .001	< .001	< .001	< .001	< .001
	**p**	0.15	0.18	0.11	0.02	0.13
	**R**^**2**^ **F**	< .001	< .001	< .001	0.01	< .001
high smartphone usage time (> 2 h/d)	**b**	-1.06	-1.10	-0.16	-0.30	-1.76
	**CI**	-2.80 – 0.68	-2.90 – 0.70	-2.00 – 1.68	-2.40 – 1.80	-3.55 – 0.02
	**95%**	-0.05	-0.05	-0.01	-0.01	-0.09
	**ß**	-1.20	-1.20	-0.17	-0.28	-1.94
	**t**	0.23	0.23	0.86	0.78	0.05
	**p**	0.08	0.10	0.05	0.001	0.03
	**R**^**2**^ **F**	< .001	< .001	< .001	0.98	0.001
**Multiple Regression**						
SAPS score (PSU symptoms)	**b**	**-0.39**	**-0.45**	**-0.42**	**-0.25**	**-0.49**
	**CI**	-0.51—-0.28	-0.57 – -0.33	-0.54—-0.29	-0.40—-0.11	-0.61—-0.37
	**95%**	-0.27	-0.27	-0.27	-0.15	-0.34
	**ß**	-6.64	-7.34	-6.63	-3.46	-8.24
	**t**	< .001	< .001	< .001	< .001	< .001
	**p**					
high smartphone usage time (> 2 h/d)	**b**	0.04	0.15	1.00	0.41	-0.38
	**CI**	-1.67 – 1.75	-1.60 – 1.91	-0.81 – 2.81	-1.71 – 2.53	-2.10 – 1.34
	**95%**	0.002	0.05	0.05	0.02	-0.02
	**ß**	0.05	0.17	1.09	0.38	-0.43
	**T**	0.96	0.86	0.28	0.70	0.66
	**p**					
	**R**^**2**^	0.15	0.18	0.11	0.02	0.13
	**F**	< .001	< .001	< .001	0.03	< .001

All associations are adjusted for gender, age, SES, *b* non-standardized regression coefficient, *ß* standardized regression coefficient. Significant associations are highlighted in bold

### Associations of SAPS scores (PSU symptoms) and daily smartphone usage time with school performance

The number and percentage of children and adolescents exhibiting good performance in each school subject are presented in Table 5. Both the simple and the multiple regression showed that adolescents reporting higher SAPS scores were significantly more likely to have poor school grades in German (OR = 1.04, *p* < 0.01) and in the first foreign language (OR = 1.04, *p* < 0.01). Associations with school grades in physical education and mathematics were significant in the simple (both OR = 1.03, *p* < 0.05), but not the multiple regression analyses (see Table 5). For children and adolescents with lower SAPS scores (SAPS score of 22, 25^th^ percentile), the likelihood of poor school grades in German and the first foreign language were 29% and 33%, compared to 38% and 42% for children and adolescents with higher SAPS scores (SAPS score of 32, 75^th^ percentile). Smartphone usage times > 2 h/d, in contrast, were not significantly associated with school performance in either the simple or multiple regression analysis (the only exception is a significant association with physical education in the simple regression) (see Table 5).

**Table 5:** Associations of SAPS scores (PSU symptoms) and high smartphone usage time (> 2 h/d) with school performance (*N* = 564)

school performance in						
		**physical education**		**German**	**mathematics**	**first foreign language**
N (%) good		433 (77%)		360 (64%)	269 (48%)	343 (61%)
**Simple regression**						
SAPS score (PSU symptoms)	**OR**	**1.03**		**1.04**	**1.03**	**1.04**
	**CI 95%**	1.004—1.07		1.01–1.07	1.001 – 1.06	1.01–1.07
	**p**	0.03		0.004	0.04	0.004
high smartphone usage time (> 2 h/d)	**OR**	**1.66**		1.31	1.22	1.40
	**CI 95%**	1.01 – 2.72		0.86 – 2.00	0.83 – 1.80	0.93 – 2.10
	**p**	0.04		0.20	0.30	0.10
**Multiple regression**						
SAPS score (PSU symptoms)	**OR**	1.03		**1.04**	1.03	**1.04**
	**CI 95%**	0.99 – 1.06		1.01 – 1.07	0.99 – 1.05	1.01 – 1.07
	**p**	0.06		0.007	0.06	0.01
high smartphone usage time (> 2 h/d)	**OR**	1.52		1.17	1.14	1.26
	**CI 95%**	0.92 – 2.52		0.76 – 1.81	0.77– 1.69	0.83 – 1.92
	**p**	0.10		0.46	0.51	0.27

All associations are adjusted for gender, age, SES; OR = odds ratio. Significant associations are highlighted in bold

## Discussion

In the present sample, more than half of participants spent more than 2 h per day on their smartphone and therefore exceeded the recommendation to limit screen time to a maximum of 2 h per day [48, 49]. However, with only 2.3% of participants scoring above the clinical cut-off of 42, the presence of (clinically relevant) PSU was low. In a Korean adolescent sample completing the same questionnaire, PSU was present in 31% of participants [13]. Moreover, the amount of PSU symptoms was lower in the present (mean = 27.8) than in the Korean sample (mean = 33.67) [13]). These results confirm our expectations that PSU symptoms are less frequent among German children and adolescents than among their Asian peers. Possible reasons are cultural differences and differences in the digitalisation process [8]. Surprisingly, the amount of PSU was also much lower than in previous European studies (9% in Spain [37] and 10% in UK [38]) However, these studies used other measurement methods, therefore, comparisons are only possible to a limited extent. High smartphone usage time was significantly associated with more PSU symptoms, which is in line with previous study findings [13, 36, 56] and our hypothesis. With respect to different smartphone activities, our findings suggest that intensive smartphone use for entertainment purposes (e.g., watching video clips, social networking or playing games), unlike use for information-seeking or text messaging, plays a major role in the development of addiction symptoms. These findings are in agreement with our assumptions and other studies, which also identified social networking and gaming as predictors for PSU [13, 41, 56]. This finding confirms the assumption that social networking and playing computer games have a particularly addictive potential, e.g., because these activities strongly hook children's and adolescents' attention and increase the psychophysiological state of arousal, e.g. due to the pleasure of playing or constant social comparisons with others [14, 40, 41]. The findings showed that among children and adolescents, the amount of PSU symptoms increases with age. This is in line with findings from other studies [14, 57] and confirms our hypothesis. Increasing peer pressure and “fear of missing out” (FOMO) might explain this association [38, 58]. In line with our hypothesis and previous studies, female gender was significantly associated with PSU symptoms. This might be explained by more intensive use of social networking sites in girls [17, 59, 60]. As seen in our findings and other studies, social networking plays a major role in the development of PSU symptoms [41]. In line with previous studies on media use [16, 22, 23], we observed higher daily smartphone usage times in children and adolescents with lower SES. However, SES was not significantly associated with PSU symptoms, indicating that PSU symptoms occur in all social classes, as expected. This finding suggests that other environmental and personal factors might be more essential in predicting PSU than social factors or factors related to the family environment. Our analyses indicate that children and adolescents who report more PSU symptoms also report more behavioural difficulties, a lower QoL, and poorer school performance. All associations reached statistical significance confirming our hypotheses. Also, nearly all associations remained significant after adjusting for the duration of participants’ daily smartphone use. The finding that PSU was associated with both internalizing (emotional problems, peer-relationship problems) and externalizing behavioural difficulties (conduct problems and hyperactivity/inattention) might be explained by different media activities related to PSU. As shown in the present study, social networking but also playing computer games or watching video clips showed the strongest associations with PSU symptoms. In previous studies, social networking was shown to be associated with internalizing behavioural difficulties [29, 61], while intensive gaming was also shown to be associated with externalizing behavioural difficulties [62, 63]. In contrast to PSU symptoms, the duration of smartphone use was only significantly associated with emotional and conduct problems and school performance in physical education. The analyses revealed no significant associations with QoL or performance in the other school subjects. This contradicts other studies showing associations between smartphone use (without considering PSU symptoms) and psychological and behavioural problems [34, 64]. Taken together, our findings suggest that it is not duration of use per se, but lower daily functioning due to smartphone use that is associated with behavioural difficulties, lower QoL, and poorer school performance. By the time adolescents realize that they have lost control of their smartphone use, that they need their smartphone to feel good, and that their smartphone use may affect their social life and their ability to concentrate on other things, they already show behavioural or academic problems. These findings support the displacement theory, which states that high media usage displaces other activities, e.g., social, physical, or academic activities, which might lead to problems in these fields [29]. The exact causes of the behavioural difficulties identified here needs to be investigated in further studies. Possible reasons could be violent or age-inappropriate content as well as peer victimization or addictive behaviour towards social networking sites [61]. Lower school satisfaction and learning comprehension could also be explained by a decreased ability to concentrate due to PSU symptoms, e.g. intensive engagement in social networking sites, constant thoughts about one’s smartphone or FOMO [65, 66]. However, it must be noted that causality is not clearly established due to the cross-sectional nature of the study.

### Strength and limitations

The strengths of this study are its focus on smartphone use in a large German sample of children and adolescents, its distinction between PSU symptoms and smartphone usage duration, and its consideration of different smartphone activities. One limitation is the high SES of the participating families and thereby the underrepresentation of socially disadvantaged children. This limits the sample’s representativeness. When dividing daily smartphone usage time into "normal" and "high", the 2 h recommended by current guidelines were taken as a benchmark, but it should be noted that even a "normal" amount of screen time might not necessarily be beneficial to health. Also, considering smartphone usage time alone does not take into account what exactly children and adolescents do on their smartphones. Other limitations are the cross-sectional nature of the study, the use of self-report questionnaires as well as the fact that behavioural problems and QoL were only recorded using (screening) questionnaires. We did not consider mental illnesses such as depression, attention deficit disorder, or anxiety.

## Conclusion

The study findings suggest that intensive smartphone use for entertainment (especially social networking), but not use for information-seeking, increases the risk of developing PSU symptoms in children and adolescents. Furthermore, the results indicate that PSU symptoms (more than long smartphone usage times per se) are associated with a lower QoL, more behavioural difficulties, and poorer school performance. From first use, children and adolescents should be made aware of the potential dangers of high smartphone use and be supported in developing and understanding of the different dependency risks lurking behind different user behaviours. This might be achieved through school education programs or training opportunities for parents. Furthermore, smartphones should not be used to solve or suppress (mental) problems. Initial PSU symptoms should be recognized and taken seriously.

## Data Availability

The datasets generated and/or analysed during the current study are not publicly available due to ethical restrictions. The LIFE Child study is a study collecting potentially sensitive information. Publishing data sets is not covered by the informed consent provided by the study participants. Furthermore, the data protection concept of LIFE requests that all (external as well as internal) researchers interested in accessing data sign a project agreement. Researchers that are interested in accessing and analysing data collected in the LIFE Child study may contact the data use and access committee (forschungsdaten@medizin.uni-leipzig.de).

## Abbreviations

FOMO: Fear of missing out
PSU: Problematic smartphone use
QoL: Quality of life
SES: Socio-economic status
SAPS: Smartphone Addiction Proneness Scale
